# Developing the draft descriptive system for the child amblyopia treatment questionnaire (CAT-Qol): a mixed methods study

**DOI:** 10.1186/1477-7525-11-174

**Published:** 2013-10-22

**Authors:** Jill Carlton

**Affiliations:** 1 Health Economics and Decision (HEDS), School of Health and Related Research (ScHARR), University of Sheffield, Regent Court, 30 Regent Street, Sheffield S1 4DA, UK

**Keywords:** Patient reported outcomes, Quality of life, Child, Paediatric, Questionnaire, Amblyopia

## Abstract

**Background:**

Amblyopia is a visual condition that occurs in childhood. Screening programmes exist within the United Kingdom (UK) to detect amblyopia, and once detected treatment is given.

Existing patient reported outcome (PRO) measures for amblyopia do not meet current recommendations for the methods adopted during their development, or the way in which the instruments are administered. The overall aim of this study was to produce a self-complete PRO instrument for amblyopia for children aged 4–7 years that uses children’s responses in the development phase. The study comprised a number of stages. This paper reports on the refinement of the descriptive system for the draft instrument (the Child Amblyopia Treatment Questionnaire, CAT-QoL) using qualitative and quantitative methods.

**Methods:**

The study consisted of three components. Children were asked to read, and complete the draft questionnaire as independently as possible. They were then asked about the questionnaire, and its format, in a cognitive debriefing exercise. Observations were made as to the child’s ability to read the questionnaire, particular attention was made as to which individual words participants struggled to read. Children were also asked their opinion on the design layout of the questionnaire. Finally, some children were asked to complete a ranking task to help determine the order of the levels of the items as judged by the children. Mid-rank scoring and statistical level of agreement were calculated for the ranking exercise.

**Results:**

Thirty-two (n=32) participants completed a draft questionnaire; each of these underwent a cognitive de-briefing interview. Twenty-two (n=22) children completed the ranking exercise. Ten children did not understand the concept of ranking. The results of the qualitative phase (cognitive de-briefing interview) were used to modify the wording of items and layout of the instrument. Results of the ranking exercise were used to inform the order of the response levels for the items.

**Conclusion:**

Responses of young children can be used in the development of PRO instruments. They are able to help inform the content, wording, and format of an instrument, ensuring good content and face validity. The results have been used to further refine the CAT-QoL, however further research is required to assess the psychometric properties of the instrument.

## Introduction

The use of patient-reported outcome (PRO) measures is increasing. They provide information which may be used to aid clinical decision making or resource allocation. New PRO instruments are being developed, or modified, to assess the impact of a condition or disease. With such an influx of instruments available to researchers and clinicians, it can be difficult to determine which instrument is most appropriate. The COSMIN initiative (COnsensus-based Standards for the selection of health Measurement INstruments) was developed to improve the selection of health measurements. The COSMIN checklist was developed through an international Delphi study, and specifically addressed the lack of agreement between terminology and definition of measurement properties [1]. These included the three domains of reliability, validity and responsiveness, all of which should be evaluated when assessing the psychometric properties of a PRO instrument. Traditionally, the psychometric assessment of PRO instruments has been reported in the literature, with little or no reference to the development of the instrument itself. However recent recommendations from the US Department of Health and Human Services Food and Drug Administration (FDA) are that there should be evidence from qualitative studies to demonstrate that the items of an instrument are appropriate to the target population, and advocate instrument development history to be available [2]. They state that the purpose of a PRO instrument is “to capture the patient experience, an instrument will not be a credible measure without evidence of its usefulness from the target population of patients” [2].

Amblyopia is a visual condition that occurs in childhood. Screening programmes exist within the United Kingdom (UK) to detect amblyopia, and once detected treatment is given. Treatment primarily consists of glasses, patch, drops, or any combination of these. There are advantages and disadvantages to the different treatment options, and these may influence treatment choice. Due to the maturing nature of the visual system, treatment is more successful if completed before the age of 7 years. This falls within an important time of a child’s personal, social and educational development. A systematic review of the literature showed that amblyopia and/or its treatment does affect an individual’s health-related quality of life (HRQoL) [3]. The ways in which these were reported varied, both in terms of the instruments used, and the respondent. Few of the studies identified in the review reported from the patient’s (child’s) perspective. Although proxy-reported PRO measures are used in healthcare, they do have limitations. They may contain items (questions) that require a parent/guardian to make a judgement on what their child is experiencing. This may not be the same as what the child is experiencing. A parent’s judgement may be influenced by how important they perceive the activity to be (such as school work, or interacting with friends); or how important they judge the treatment itself. Furthermore, parental reporting can introduce potential bias based upon how the disease impacts upon the parent themselves [4]. Two disease-specific PRO instruments were identified in the literature review that could be used to assess the HRQoL implications of amblyopia the Amblyopia and Strabismus Questionnaire (A&SQ) and the Amblyopia Treatment Index (ATI) [5]. Both instruments were designed specifically for the amblyopic population and have been accepted within the clinical community as being valid and reliable [6-9]. The A&SQ and ATI were developed using a “top-down” methodological approach; that is, the content of the instrument was determined by previous literature and clinical opinion. A disadvantage of the A&SQ is that it is administered to adults, so cannot be used to assess the implications of amblyopia and/or its treatment from the child’s perspective. Furthermore, it contains questions that relate to strabismus. Strabismus is an abnormal alignment of one or both eyes (also known as a squint), and has different HRQoL implications to amblyopia. The ATI is more likely to be sensitive to encapsulating the HRQoL implications of amblyopia, as it does not address issues of strabismus (unlike the A&SQ); yet this instrument also has some potential weaknesses when considering its application to the paediatric population. It is a proxy-reported instrument, and was initially designed for that purpose. However, in a recent study the Pediatric Eye Disease Investigator Group (PEDIG) used the ATI to assess the impact of treatment in children aged 7–13 years [10]. Parents and children both completed the ATI and the results were compared. The psychometric properties of the two ATI versions were reported to be similar, however the authors acknowledged that the validity of the child version has yet to be formally been assessed.

No disease-specific PRO instrument is available to assess the implications of amblyopia treatment from the child’s perspective that satisfies the recommendations of the FDA and COSMIN Initiative. The overall aim of this study was to produce a paediatric disease-specific health related quality of life (HRQoL) instrument for amblyopia that could be used in research or routine clinical practice. The study comprised a number of phases, the results of which have been reported. Firstly, a systematic literature review was undertaken to identify the HRQoL implications of amblyopia and/or its treatment [3]. The results of the literature review were used to create a topic guide used in focus groups conducted with clinicians (orthoptists) [11]. The focus group sessions were undertaken to identify any additional HRQoL implications of amblyopia and/or its treatment which had not been identified in the literature review. The results of the literature review and focus group sessions were used to create a topic guide for semi-structured interviews conducted with children with amblyopia. The interviews identified potential themes that could be used in a paediatric disease-specific HRQoL instrument for amblyopia [12]. This paper reports on the refinement of the descriptive system for the draft questionnaire (the Child Amblyopia Treatment Questionnaire, CAT-QoL) using qualitative and quantitative methods.

## Methods

Eleven possible themes had been identified for possible inclusion in the draft instrument following qualitative analysis of semi-structured interviews conducted with children with amblyopia [12]. These are listed in Table 1. Analysis of the interview data identified potential themes for inclusion in the CAT-QoL instrument; however, the response levels for the items (questions) had not yet been determined. The interview transcripts were re-examined and the terminology the children used when describing the impact of amblyopia and/or its treatment upon their HRQoL informed the choice of levels for the items (questions). The phrases that the children used were *“a little bit”*; *“a bit”*, *“quite a bit”*; *“quite”*; *“a lot”*; *“really”*; and *“very”*. The transcripts were re-examined to establish which phrases had been used by the children interviewed for each of the given items shown in Table 1. Not every phrase was used when the children discussed their feelings about each possible item, and therefore three categories of questions (A, B and C) with different item response scales were established (Table 1). The item response scales were applied to each of the 11 items, to form an 11-item instrument that consisted of six item response levels.

**Table 1:** Potential item for the CAT-QoL instrument

**1**	**Item**	**Category**
2	Physical sensation of the treatment (e.g. feeling of the patch/glasses on the face, or the feeling of the drops being instilled)	A
3	Pain of treatment (hurt)	A
4	Being able to play with other children	A
5	How other children have treated them (like laughing or name-calling)	A
6	Ability to undertake work at school	B
7	Ability to undertake other tasks (like playing on the computer, colouring, playing games, watching TV)	B
8	Feeling sad or unhappy	B
9	Feeling cross	B
10	Feeling worried	B
11	Feeling frustrated	B
12	Feelings towards family members (like parents or siblings)	C

A not; a little bit; a bit; quite a bit; a lot; really.

B not; a little bit; a bit; quite; really; very.

C not; a little bit; a bit; quite a bit; really, very.

Two draft versions of the questionnaire were developed. These were the same in content but differed in format. One version had the completion tick-box at the start of the item statement (version 1). The other version had the completion tick-box at the end of the item statement (version 2). Seven different treatment-specific versions were created (patch; drops; glasses; patch and drops; patch and glasses; glasses and drops; glasses, patch and drops). It was necessary to have treatment-specific versions to word the questions appropriately.

The 11-item draft questionnaires were piloted in a clinical setting at two National Health Service (NHS) sites within Sheffield, United Kingdom (UK). The purpose of the pilot was to refine the content, wording and format of the CAT-QoL instrument, before a multi-centre validation study. The pilot consisted of three components and involved: completing a draft questionnaire; cognitive debriefing interview; and ranking exercise. Children were asked to read, and complete the draft questionnaire as independently as possible. The children were then asked about the questionnaire, and its format, in a cognitive debriefing exercise. Finally, some children were asked to complete a ranking task to help determine the order of the levels of the items as judged by the children. Each participant was given either Version 1 (tick-box at the end of the item statement) or Version 2 (tick-box at the start of the item statement) to complete.

### Cognitive debriefing

Children were also asked to participate in a cognitive debriefing process. Cognitive debriefing is a process whereby participants are asked to explain their thinking, or understanding of a question. The process is used in the development of questionnaires to help identify and correct problems with survey questions [13]. Within this study, participants were asked to explain what they believed the question was asking them. The results were recorded, and clarification sought where applicable. Observations were also made as to the child’s ability to read the questionnaire itself. Particular attention was made as to which individual words the participants struggled to read. Children were also asked their opinion on the design layout of the questionnaire, specifically whether the item check-box should be placed at the beginning or the end of each level response (Version 1 vs. Version 2) (Additional file 1).

### Example of item check-box location

Version 1

□ My patch did *not* hurt me.

Version 2

My patch did *not* hurt me… … … … … … … … … …□

### Ranking exercise to determine the order of item response levels

Qualitative analysis of the interview data identified the possible item response levels to be used in the instrument. However, it was important to determine the order of the response levels for the content of the questionnaire from a child’s perspective. To achieve this, a ranking exercise was undertaken. Ranking involves participants ordering a list of statements starting from least (affected) to worst (affected). The item response levels are printed on separate cards, and participants are asked to place these in an order that they believe to be correct. Ideally, participants would rank the order of response levels for every item within a questionnaire. However, it was felt that it would be too lengthy a process to ask a child to rank the order of levels for each of the 11 possible items in the draft questionnaire. Instead, one question was chosen from each of the categories (A, B and C). The questions chosen were “hurt”, “cross” and “upset with family”. The items “hurt” (Question 3) and “cross” (Question 5) were chosen as it was felt that these were easy words for participants to read. There was only one Category C question (Question 9). Participants were asked to rank the order of severity from least to worst. When a card was ranked first, it was scored 1; when ranked second it was scored 2; and so on. Where cards were ranked as being equal, each tied ranking was given a value of the midpoint as the previous two marks (mid rank method) [14]. This approach ensures that the sum of the ranks is maintained.

The mean rank order was calculated for each response level, for each of the category questions (A, B and C). The difference between the mean rank scores was then calculated. A small difference between the mean rank scores of the item response levels indicates that the two levels are thought of as being the same conceptually. A mean rank value of less than 0.2 was taken to mean a small difference between response levels. This approach was in line with that adopted by Stevens during the development of the Child Health Utility 9D (CHU9D) questionnaire (a generic paediatric HRQoL instrument) [15]. The rank data was also analysed by using Kendall’s coefficient of concordance test statistic. This is a measure of agreement between variables, and is recorded as a value between 0 (no agreement) and 1 (complete agreement). The Kendall’s coefficient of concordance test statistic measured the amount of agreement between participants on how they ranked the order of the response levels for the questions used in the ranking task. Landis and Koch use categories to define levels of agreement (for the Kappa statistic, another statistical measure of agreement) [16]. Each question was assessed in terms of the level of agreement (*Poor* < 0; *Slight* 0.00 – 0.20; *Fair* 0.21 – 0.40; *Moderate* 0.41 – 0.60; *Substantial* 0.61 – 0.80; and *Almost Perfect* 0.81 – 1.00).

The study was approved by the NHS Research Ethics Committee (REC Ref: 07/Q1201/5), and followed the tenets of the Declaration of Helsinki. The inclusion criteria were that adopted for the semi-structured interviews, and the same recruitment technique was adopted [12]. It was not possible to identify before clinical examination potential participants, and therefore purposive sampling of the eligible study population was not possible. Instead, an opportunistic recruitment (and therefore opportunistic sampling) approach was undertaken. Full written consent was obtained from the parent/guardian before the child participated in the study.

## Results

Thirty-two (n=32) participants completed a draft questionnaire, and each of these underwent a cognitive de-briefing interview. All of the participants in the pilot sample were white. The majority of the participants (n=26) had mild level of amblyopia (using the PEDIG model of amblyopia classification) [17-21]. Mild amblyopia was categorised as 0≥0.30 logMAR; moderate amblyopia 0.31≥0.60 logMAR; and severe amblyopia >0.61 logMAR). Only 3 participants had a moderate level of amblyopia; the remaining 3 participants did not have their vision assessed at the time of the pilot, and therefore their level of amblyopia severity could not be classified. Eleven participants completed version 1 of the questionnaire, and 19 participants were issued with version 2. Of the 32 participants, all were on some form of treatment (glasses only n=13; patch only n=1; patch and glasses n=16; glasses and drops n=2)). Of the study sample, 22 children were able to complete the ranking exercise (Table 2). Ten children did not understand the concept of the ranking exercise, and this exercise was abandoned for these participants.

**Table 2:** Study participants for pilot of draft questionnaire

	**N**	**Sex**	**Number of participants age (yrs)**				
			**5**	**6**	**7**	**8**	**9**
Completed Questionnaire and Cognitive Debriefing	32	16 male, 16 female	14	8	5	4	1
Version1	11	5 male, 6 female	7	2	1	1	0
Version 2	19*	10 male, 9 female	8*	6	3	1	1
Able to self-complete			4	1	5	3	1
Questionnaire read to them			9	4	0	1	0
Questionnaire read to them due to drops			1	3	0	0	0
Ranking Exercise	22	11 male, 11 female	8	4	5	4	1
Unable to complete ranking	10	5 male, 5 female	6	4	0	0	0

* abandoned in one participant.

### Practicality of the instrument

Of the 32 children who took part in the study, 29 completed the questionnaire. Two children did not complete the entire questionnaire due to time constraints (n=2). One questionnaire was abandoned as the child was unresponsive (n=1). Of the 29 children who completed the questionnaire, 13 were able to read the questionnaire themselves (either in its entirety or with limited assistance). A number of children had to have the questionnaire read to them, as they had had drops in their eyes for a routine glasses check (n=5). This meant their vision was blurry and they were unable to read the text itself. It is not certain whether these children would have been able to self-complete the questionnaire if the drops had not been instilled. The remaining 11 children had the questionnaire read to them. This was mainly at their request. Of the children who completed the questionnaire themselves (n=13), each responded to every question, giving a 100% completion rate.

### Content validity and face validity

As part of the cognitive de-briefing process, each child was asked if there were any additional questions that should be included. They were asked whether there was “*anything else*” that they could not do because of their specific eye treatment; and whether they had any other feelings about their eye treatment that had not been asked. One child said that they “*bumped into things*” because of their patch and glasses. Another said that their patch and glasses felt “*tickly*”, and that their patch and glasses made them feel “*happy*”. One child said that they “*couldn’t sit far away from the whiteboard at school*” because of their patch and glasses. Another child said that they felt “*annoyed*” due to their treatment. Some of the children needed some assistance reading particular words. These included “*worried*”, “*frustrated*” (annoyed, cross), “*laughing*”, “*question*”, “*each*”, “*choices*”, “*yesterday*”, and “*bothered*”. Some children needed the meaning of some questions explained to them. These questions were “*feeling worried*”, “*upset with my family*”, “*feeling frustrated*”.

### Ranking exercise

The results for the mean rank, standard deviation, minimum and maximum position for each question are shown in Table 3. Table 4 shows the mean rank order and the difference in mean rank order between the levels. A small difference between the ranking scores indicates that the participants view the two levels to have a similar meaning. In the category A question (*hurt*) there is little difference between “*my drops hurt me a lot*” and “*my drops really hurt me*” to be the same (as shown by a difference of 0.23). The results show that within the category B question (*cross*), “*my drops made me feel really cross*” and “*my drops made me feel very cross*” were valued as being the same (difference of -0.09). The negative value indicates that “*very*” can be placed above “*really*”. Table 5 shows the Kendall coefficient for each question. The Kendall coefficient is “substantial” for the category A (*hurt*) and category C (*upset with my family*) questions, and “almost perfect” for the category B (*cross*) question (using Landis & Koch definitions [16]). This indicates that children are able to rank the order of the levels, and that statistically, the ordering of the levels (as shown in Table 3) is “correct”.

**Table 3:** Mean rank, standard deviation, minimum and maximum position for each question

	**Question category**	**Mean rank**	**SD**	**Min**	**Max**
My drops did *not* hurt me	A	1.00	0.00	1	1
My drops hurt me *a little bit*	A	2.45	0.77	1.5	4
My drops hurt me *a bit*	A	3.00	0.65	1.5	4
My drops hurt me *quite a bit*	A	3.59	0.77	2	4
My drops hurt me *a lot*	A	5.36	0.52	4.5	6
My drops *really* hurt me	A	5.59	0.43	4.5	6
My drops did *not* make me feel cross	B	1.05	0.21	1	2
My drops made me feel *a little bit* cross	B	2.36	0.71	1	4
My drops made me feel *a bit* cross	B	2.89	0.55	2	3.5
My drops made me feel *quite* cross	B	3.70	0.63	2	4
My drops made me feel *really* cross	B	5.55	0.41	5	6
My drops made me feel *very* cross	B	5.45	0.41	5	6
My drops have *not* made me get upset with my family	C	1.00	0.00	1	1
My drops have made me get upset with my family *a little bit*	C	2.50	0.71	2	4
My drops have made me get upset with my family *a bit*	C	2.91	0.72	2	5
My drops have made me get upset with my family *quite a bit*	C	3.73	0.83	2	6
My drops have *really* made me get upset with my family	C	5.20	0.70	3	6
My drops have made me get *very* upset with my family	C	5.61	0.43	5	6

**Table 4:** Mean rank order and the difference in mean rank order between the levels

	**Mean rank order**	**Difference**
My drops did *not* hurt me	1.00	1.45
My drops hurt me *a little bit*	2.45	0.55
My drops hurt me *a bit*	3.00	0.59
My drops hurt me *quite a bit*	3.59	1.77
My drops hurt me *a lot*	5.36	0.23
My drops *really* hurt me	5.59	
My drops did *not* make me feel cross	1.05	1.32
My drops made me feel *a little bit* cross	2.36	0.52
My drops made me feel *a bit* cross	2.89	0.82
My drops made me feel *quite* cross	3.70	1.84
My drops made me feel *really* cross	5.55	-0.09
My drops made me feel *very* cross	5.45	
My drops have *not* made me get upset with my family	1.00	1.50
My drops have made me get upset with my family *a little bit*	2.50	0.41
My drops have made me get upset with my family *a bit*	2.91	0.82
My drops have made me get upset with my family *quite a bit*	3.73	1.48
My drops have *really* made me get upset with my family	5.20	0.41
My drops have made me get *very* upset with my family	5.61	

**Table 5:** Kendall coefficient for questions

**Question**	**Kendall coefficient**
Category A (*hurt*)	0.795
Category B (*cross*)	0.836
Category C (*upset with my family*)	0.755

### Modifications to draft CAT-QoL instrument

A number of modifications were made to the initial questionnaire driven by the results of the cognitive de-briefing and ranking exercises. The main alterations are discussed here. Firstly, the results of the ranking data analysis were used to determine both the number of levels and order of the levels for the items. Despite there being a *possibility* that participants believe “*my drops hurt me a lot*” and “*my drops really hurt me*” to be thought of as the same (as shown by a difference of 0.23), category A questions were kept as a 6-part response scale. However, all category B questions were reduced to a 5-part response scale, with the “*really*” level removed. Table 4 shows the mean rank order, and the difference in the mean rank order between the levels. For the “cross” question, the small difference in mean rank order indicates that children could not distinguish much difference between “*really”* cross and “*very”* cross. It could be interpreted that one of these levels is redundant, and a decision was made to remove one from the draft instrument. “*Really*” was chosen as it was felt this could be considered a colloquial term. When this response option was removed, this 5 response level options remained: “*did not”*, “*a little bit”*, “*a bit”*, “*quite”*, “*very”*. As “*cross*” was a Category B question, all other category B questions were revised so that they too had 5 response levels.

The wording of one question was modified (“*upset with my family*”) due to comments during the cognitive de-briefing. Participants reported that they *did* feel upset due to their treatment, but not necessarily upset with their family. The interview transcripts were re-examined to consider this. It can be argued that “*upset*” should be used as an item; just as “*sad*” is. The items are the consequence of the treatment, and not a description of why they feel that emotion. The question was therefore changed from “*My patch has not made me get upset with my family*” to “*My patch has not made me feel upset*”. Once the “*with my family*” was removed, and the term “*feel*” rather than “*get*” applied, some of the original response levels no longer made sense (“*My drops have made me feel quite a bit upset*”). And so the “a bit” was dropped, leaving “*quite”*. This meant the response options for the question were “*did not”*, “*a little bit”*, “*a bit”*, “*quite”*, “*really*”, “*very”*; which were the same as that of the Category B question, and therefore the Category C question was re-categorised to B. The responses options Category B questions were modified in response to the difference in mean rank order as outlined above, and therefore 5 response level options were applied.

One question was omitted in its entirety from the draft questionnaire (“*feeling frustrated*”). The majority of participants in the cognitive de-briefing had to have the concept of “*frustration*” explained to them. Even after an explanation was given, some children still did not understand what was meant by the term. One additional question was included in the draft questionnaire (“*happy*”). As part of the de-briefing process, participants were asked if they felt anything else about their patch, drops and/or glasses. A number of children reported that they felt happy. The transcripts of the interviews with children were then re-examined, and analysed again to explore the possibility of happy as an item in the questionnaire. This had not been originally included in the draft questionnaire as it was assumed that “*happy*” was the opposite of “*sad*”. However, as the aim of this study was to develop an instrument by children, for children, the cognitive de-briefing responses were noted and this item was added.

Other minor modifications included changing individual words to make it easier for the child to understand or read; or reducing the number of words for a given question where possible. The purpose was to make the overall task easier for participants. (See Additional file 2 for modified version of questionnaire, and Additional file 3 for full details of all modifications made).

## Discussion

The literature on the development of the descriptive system for PRO instruments is relatively sparse. This research describes some of the processes undertaken to refine the draft descriptive system of the CAT-QoL instrument. This is in line with recommendations of the FDA for development of PRO measures [2]. The aim of the study was to further refine the draft descriptive system, prior to a multi-centre pilot and validation study, after which the final CAT-QoL instrument would be developed.

### Practicalities of the instrument: task burden

Language comprehension and reading ability are important factors, particularly if they are to “self-complete” the questionnaire [22]. The reading ability of the children who completed the piloting of the draft questionnaire was not assessed in this study. It is acknowledged that the degree to which a child can self-complete a questionnaire is dependent upon their ability. Matza et al. advise that younger children may require assistance with reading the questionnaire, and the administration procedures [4].

### Format of the instrument: number of items

The format of the questionnaire in terms of length, and item level scales are other issues to consider. The number of items included in a PRO instrument contributes to the response burden of the task. It is acknowledged that older children can be expected to complete longer measures [4]; with the intended target population of 4–7 year old, the draft CAT-QoL instrument could be described as having a low task burden.

### Format of the instrument: response levels – frequency or severity scales?

PRO instruments can differ in their response scale options. They may be based upon severity, frequency, or the level of agreement with something (strongly agree, strongly disagree). The severity scale was chosen for the CAT-QoL for two reasons. Firstly, the data collected during the interviews with the children lends itself to this approach. Children spoke of how bad something was, rather than how often it occurred. Secondly, using a frequency scale would involve the child having to accurately consider a given time period. Children’s abilities to reliably report on their health within specific time frames have been investigated. Predictably, older children are more accurately able to demonstrate a longer recall period [22,23]. Although a time frame has been set for the CAT-QoL instrument (“in the last week”), it was felt that using frequency item scale would result in less accurately reported data. Severity scales have been used successfully in other paediatric PROs, such as the CHU9D [15].

A time frame has been set for the CAT-QoL instrument (“in the last week”). Other paediatric PROs, such as the PedsQL^TM^, use a time frame of in the “last few weeks”. The CHU9D uses a time frame of “today”. Consideration was made of the intended population (children aged 4 to 7 years), so a time frame of the “last few weeks” was deemed too long to be able to accurately recall. A time frame of “today” was not chosen, as a potential participant may receive the CAT-QoL instrument early in the day, before they have had time to undertake their amblyopia treatment and wear their patch (or glasses, etc.).

### Format of the instrument: response levels – number of response scale points

The number of response scale points within a PRO instrument is important to consider. Matza et al. state that younger children show significantly more extreme responses than older children [4]. However, children aged 8 years have been shown to accurately use a 5-part or 7-part response scale [24]. The number of response scale points in the draft version of the CAT-QoL was informed initially from the child interview data, and revised following the pilot and ranking exercise. The results of the ranking exercise demonstrated that young children were able to rank severity in a way that appears conceptually coherent. This is a novel approach in the development of response scales options for PRO instruments for young children.

### Format of the instrument: layout

Some questionnaires, such as the PedsQL™ child report use a smiley face to illustrate the difference in response options [25]. Similarly, the Child Health and Illness Profile (CHIP) use circles of increasing size and illustrations to show the extreme responses the child can give [22]. The impact of response scale options upon reliability and reproducibility has been investigated using the TedQL instrument (a generic child self-report instrument) [26]. In a study on healthy children, Creemens et al. reported that young children (aged 5–6 years) showed better reliability (agreement in responses over time, test-retest reliability) using a thermometer type response scale. The use of pictures, or circles of increasing size, was not adopted for the CAT-QoL layout for a number of reasons. It was felt children may interpret a smiley face to be how they feel on that day, rather than a reflection of how they have felt about the item. Some children also associate sad faces with negative connotations. For example, a smiley face often denotes “happy”. A child may not answer a question on “hurt” correctly, as they may respond with a “sad” face, as they perceive “hurt” to be a negative concept. In this case, they would not be reporting upon their HRQoL. Children in this study reported that they preferred the tick-box to be at the end of the item statement (Version 2).

### Limitations of the study

This research is not without limitations. The number of participants in the pilot of the draft version of the descriptive system was small, and the number of those who participated in the ranking exercise of the response levels was smaller still. It could be argued that more participants should have been included during this phase of the instrument development. However, a larger number of children were involved in the prior phase (identification of potential items through semi-structured interviews) [12].

Furthermore, the classification of amblyopia adopted for the study is arbitrary. There are universally agreed definitions of what level of visual acuity equates to mild, moderate, and severe amblyopia. The categorisation approach used here was that described by the PEDIG group; a collaborative network who facilitate multi-centre clinical research in eye disorders that affect children. The PEDIG group have published widely in the field of amblyopia, and have conducted a number of multi-centre studies examining the efficacy of amblyopia treatment [17-21]. The majority of participants in this study had mild level of amblyopia, and it could be argued that more participants should have been included to reflect the full spectrum of amblyopia severity.

## Conclusions

The aim of this paper was to describe the methods used to refine a disease-specific HRQoL instrument designed for children aged 4–7 years with amblyopia. This research has demonstrated that young children are able to ensure face validity of a PRO. Children were used at every stage of the development of the descriptive system. Interview data directly informed the items of the instrument; the response levels of the instrument; and the wording of the instrument. Comments given by the children during the cognitive de-briefing process were also used to alter the layout and format of the measure itself. This approach ensured the content validity and face validity of the instrument are high. This adheres to the recommendations of the FDA who observe that issues for PRO instruments applied to children include “age-related vocabulary, language comprehension, comprehension of the health concept measured, and duration of recall” [2]. Further research is required to formally assess the draft descriptive system, and subsequent refinement may be necessary. The draft descriptive system outlined here is not the final content of the CAT-QoL instrument. The draft descriptive system comprised of eleven items, with either five or six severity response options. However, the final number of items, and the number of response level options for each item will be explored following a multi-centre pilot and validation study. Assessment of the psychometric properties of the final CAT-QoL instrument should also be conducted. Further details on the CAT-QoL instrument can be found at http://www.cat-qol.org.

## Competing interests

The author declares that he has no competing interests.

## Supplementary Material

Additional file 1 Child Amblyopia Treatment Questionnaire (CAT-QoL). Click here for file

Additional file 2 Patch Questionnaire. Click here for file

Additional file 3 **Table S1.** Modification to draft questionnaire (from beginning to end). Click here for file
