# Supplementary material for: Developing the draft descriptive system for the child amblyopia treatment questionnaire (CAT-Qol): a mixed methods study
Source: Health Qual Life Outcomes. 2013 Oct 22;11:174. doi: 10.1186/1477-7525-11-174 (PMC3854484; doi:10.1186/1477-7525-11-174)
Supplement: Additional file 1 — Child Amblyopia Treatment Questionnaire (CAT-QoL). [file 1477-7525-11-174-S1.docx]

**ID Number:**

Trust Logo

**Child Amblyopia Treatment Questionnaire**

**(CAT-QoL)**

|  | 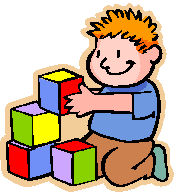 |  |
| --- | --- | --- |
| 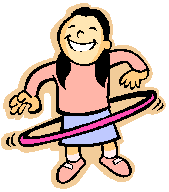 | 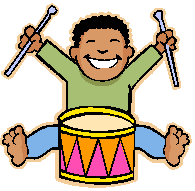 | 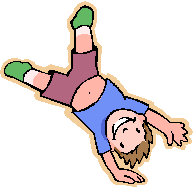 |
|  | 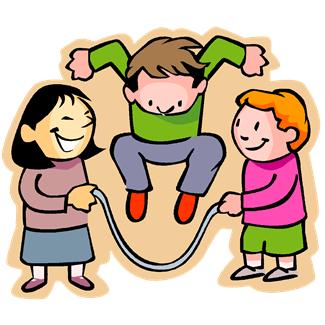 |  |

**Patch Questionnaire**

**Patch Questionnaire**

**Instructions**

These questions ask you how you have felt **in the last week**. Read each one and all of the choices, and see which one is most like you **in the last week**.

Put a tick in the box next to it, like this 🗹. Only tick one box for each question.

Yesterday I felt a bit angry because of my patch, so I will tick this box.

**Angry**

| I do not feel angry because of my patch ... ... ... ... ... ... ... ... ... ... ... ... ... ... ... ... ... ... ... | ...□ |
| --- | --- |
| I feel a little bit angry because of my patch ... ... ... ... ... ... ... ... ... ... ... ... ... ... ... ... ... ... | ...□ |
| I feel a bit angry because of my patch ... ... ... ... ... ... ... ... ... ... ... ... ... ... ... ... ... ... ... ... | ...🗹 |
| I feel a lot angry because of my patch ... ... ... ... ... ... ... ... ... ... ... ... ... ... ... ... ... ... ... ... | ...□ |
| I feel very angry because of my patch ... ... ... ... ... ... ... ... ... ... ... ... ... ... ... ... ... ... ... ... | ...□ |

| Now think about the questions below. |
| --- |

1. **Feeling of the patch on your face**

| The feel of my patch has not bothered me. ... ... ... ... ... ... ... ... ... ... ... ... ... ... ... ... ... ... | ...□ |  |  |
| --- | --- | --- | --- |
| The feel of my patch has bothered me a little bit... ... ... ... ... ... ... ... ... ... ... ... ... ... ... ... | ...□ | My patch has made me feel a little bit sad. ... ... ... ... ... ... ... ... ... ... ... ... ... ... ... ... ... ... | ...□ |
| The feel of my patch has bothered me a bit... ... ... ... ... ... ... ... ... ... ... ... ... ... ... ... ... ... | ...□ | My patch has made me feel a bit sad. ... ... ... ... ... ... ... ... ... ... ... ... ... ... ... ... ... ... ... ... | ...□ |
| The feel of my patch has bothered me quite a bit. ... ... ... ... ... ... ... ... ... ... ... ... ... ... ... | ...□ | My patch has made me feel quite sad ... ... ... ... ... ... ... ... ... ... ... ... ... ... ... ... ... ... ... ... | ...□ |
| The feel of my patch has bothered me a lot... ... ... ... ... ... ... ... ... ... ... ... ... ... ... ... ... ... | ...□ | My patch has made me feel very sad.. ... ... ... ... ... ... ... ... ... ... ... ... ... ... ... ... ... ... ... ... | ...□ |
| The feel of my patch has really bothered me. ... ... ... ... ... ... ... ... ... ... ... ... ... ... ... ... ... | ...□ |  |  |

1. **Hurt**

| My patch did not hurt me.. ... ... ... ... ... ... ... ... ... ... ... ... ... ... ... ... ... ... ... ... ... ... ... ... ... | ...□ |
| --- | --- |
| My patch hurt me a little bit... ... ... ... ... ... ... ... ... ... ... ... ... ... ... ... ... ... ... ... ... ... ... ... ... | ...□ |
| My patch hurt me a bit... ... ... ... ... ... ... ... ... ... ... ... ... ... ... ... ... ... ... ... ... ... ... ... ... ... ... | ...□ |
| My patch hurt me quite a bit. ... ... ... ... ... ... ... ... ... ... ... ... ... ... ... ... ... ... ... ... ... ... ... ... | ...□ |
| My patch hurt me a lot... ... ... ... ... ... ... ... ... ... ... ... ... ... ... ... ... ... ... ... ... ... ... ... ... ... ... | ...□ |
| My patch really hurt me.. ... ... ... ... ... ... ... ... ... ... ... ... ... ... ... ... ... ... ... ... ... ... ... ... ... ... | ...□ |

1. **Work at school (like reading and writing)**

| My patch has not made it hard to do my work at school. ... ... ... ... ... ... ... ... ... ... ... ... | ...□ |
| --- | --- |
| My patch made it a little bit hard to do my work at school.. ... ... ... ... ... ... ... ... ... ... ... | ...□ |
| My patch made it a bit hard to do my work at school.. ... ... ... ... ... ... ... ... ... ... ... ... ... | ...□ |
| My patch made it quite hard to do my work at school. ... ... ... ... ... ... ... ... ... ... ... ... ... | ...□ |
| My patch made it really hard to do my work at school ... ... ... ... ... ... ... ... ... ... ... ... ... | ...□ |
| My patch made it very hard to do my work at school... ... ... ... ... ... ... ... ... ... ... ... ... ... | ...□ |

1. **Doing other things (like playing on the computer, colouring, playing games, watching TV)**

| My patch has not made it hard to do what I want to ... ... ... ... ... ... ... ... ... ... ... ... ... ... | ...□ |
| --- | --- |
| My patch has made it a little bit hard to do what I want to.. ... ... ... ... ... ... ... ... ... ... ... | ...□ |
| My patch has made it a bit hard to do what I want to.. ... ... ... ... ... ... ... ... ... ... ... ... ... | ...□ |
| My patch has made it quite hard to do what I want to. ... ... ... ... ... ... ... ... ... ... ... ... ... | ...□ |
| My patch has made it really hard to do what I want to.... ... ... ... ... ... ... ... ... ... ... ... ... | ...□ |
| My patch has made it very hard to do what I want to.. ... ... ... ... ... ... ... ... ... ... ... ... ... | ...□ |

1. **Sad**

| My patch has not made me feel sad... ... ... ... ... ... ... ... ... ... ... ... ... ... ... ... ... ... ... ... ... | ...□ |
| --- | --- |
| My patch has made me feel a little bit sad. ... ... ... ... ... ... ... ... ... ... ... ... ... ... ... ... ... ... | ...□ |
| My patch has made me feel a bit sad. ... ... ... ... ... ... ... ... ... ... ... ... ... ... ... ... ... ... ... ... | ...□ |
| My patch has made me feel quite sad... ... ... ... ... ... ... ... ... ... ... ... ... ... ... ... ... ... ... ... | ...□ |
| My patch has made me feel really sad... ... ... ... ... ... ... ... ... ... ... ... ... ... ... ... ... ... ... ... | ...□ |
| My patch has made me feel very sad. ... ... ... ... ... ... ... ... ... ... ... ... ... ... ... ... ... ... ... ... | ...□ |

1. **Cross**

| My patch did not make me feel cross.. ... ... ... ... ... ... ... ... ... ... ... ... ... ... ... ... ... ... ... ... | ...□ |
| --- | --- |
| My patch made me feel a little bit cross.. ... ... ... ... ... ... ... ... ... ... ... ... ... ... ... ... ... ... ... | ...□ |
| My patch made me feel a bit cross.. ... ... ... ... ... ... ... ... ... ... ... ... ... ... ... ... ... ... ... ... ... | ...□ |
| My patch made me feel quite cross. ... ... ... ... ... ... ... ... ... ... ... ... ... ... ... ... ... ... ... ... ... | ...□ |
| My patch made me feel really cross ... ... ... ... ... ... ... ... ... ... ... ... ... ... ... ... ... ... ... ... ... | ...□ |
| My patch made me feel very cross.. ... ... ... ... ... ... ... ... ... ... ... ... ... ... ... ... ... ... ... ... ... | ...□ |

1. **Worried**

| My patch has not made me feel worried ... ... ... ... ... ... ... ... ... ... ... ... ... ... ... ... ... ... ... | ...□ |
| --- | --- |
| My patch has made me feel a little bit worried.. ... ... ... ... ... ... ... ... ... ... ... ... ... ... ... ... | ...□ |
| My patch has made me feel a bit worried.. ... ... ... ... ... ... ... ... ... ... ... ... ... ... ... ... ... ... | ...□ |
| My patch has made me feel quite worried. ... ... ... ... ... ... ... ... ... ... ... ... ... ... ... ... ... ... | ...□ |
| My patch has made me feel really worried. ... ... ... ... ... ... ... ... ... ... ... ... ... ... ... ... ... ... | ...□ |
| My patch has made me feel very worried... ... ... ... ... ... ... ... ... ... ... ... ... ... ... ... ... ... ... | ...□ |

1. **Frustrated**

| My patch has not made me feel frustrated. ... ... ... ... ... ... ... ... ... ... ... ... ... ... ... ... ... ... | ...□ |
| --- | --- |
| My patch has made me feel a little bit frustrated... ... ... ... ... ... ... ... ... ... ... ... ... ... ... ... | ...□ |
| My patch has made me feel a bit frustrated... ... ... ... ... ... ... ... ... ... ... ... ... ... ... ... ... ... | ...□ |
| My patch has made me feel quite frustrated.. ... ... ... ... ... ... ... ... ... ... ... ... ... ... ... ... ... | ...□ |
| My patch has made me feel really frustrated. ... ... ... ... ... ... ... ... ... ... ... ... ... ... ... ... ... | ...□ |
| My patch has made me feel very frustrated... ... ... ... ... ... ... ... ... ... ... ... ... ... ... ... ... ... | ...□ |

1. **How you have felt about your family (like your Mummy, Daddy, brother or sister)**

| My patch has not made me get upset with my family... ... ... ... ... ... ... ... ... ... ... ... ... ... | ...□ |
| --- | --- |
| My patch has made me get upset with my family a little bit ... ... ... ... ... ... ... ... ... ... ... | ...□ |
| My patch has made me get upset with my family a bit ... ... ... ... ... ... ... ... ... ... ... ... ... | ...□ |
| My patch has made me get upset with my family quite a bit.. ... ... ... ... ... ... ... ... ... ... | ...□ |
| My patch has really made me get upset with my family... ... ... ... ... ... ... ... ... ... ... ... ... | ...□ |
| My patch has made me get very upset with my family. ... ... ... ... ... ... ... ... ... ... ... ... ... | ...□ |

1. **Playing with other children**

| My patch has not stopped me playing with my friends. ... ... ... ... ... ... ... ... ... ... ... ... ... | ...□ |
| --- | --- |
| My patch has stopped me playing with my friends a little bit... ... ... ... ... ... ... ... ... ... ... | ...□ |
| My patch has stopped me playing with my friends a bit... ... ... ... ... ... ... ... ... ... ... ... ... | ...□ |
| My patch has stopped me playing with my friends quite a bit. ... ... ... ... ... ... ... ... ... ... | ...□ |
| My patch has really stopped me playing with my friends.. ... ... ... ... ... ... ... ... ... ... ... ... | ...□ |
| My patch has stopped me playing with my friends a lot .. ... ... ... ... ... ... ... ... ... ... ... ... | ...□ |

1. **How other children have treated you (like laughing at you, or calling you names)**

| Children have not laughed at me or called me names because of my patch. ... ... ... | ...□ |
| --- | --- |
| Children have laughed at me or called me names a little bit because of my patch... | ...□ |
| Children have laughed at me or called me names a bit because of my patch... ... ... | ...□ |
| Children have laughed at me or called me names quite a bit because of my patch. | ...□ |
| Children have really laughed at me or called me names because of my patch.. ... ... | ...□ |
| Children have laughed at me or called me names a lot because of my patch... ... ... | ...□ |
