# Supplementary material for: Developing the draft descriptive system for the child amblyopia treatment questionnaire (CAT-Qol): a mixed methods study
Source: Health Qual Life Outcomes. 2013 Oct 22;11:174. doi: 10.1186/1477-7525-11-174 (PMC3854484; doi:10.1186/1477-7525-11-174)
Supplement: Additional file 2 — Patch Questionnaire. [file 1477-7525-11-174-S2.docx]

**ID Number:**

Trust Logo

**Child Amblyopia Treatment Questionnaire**

**(CAT-QoL)**

|  | 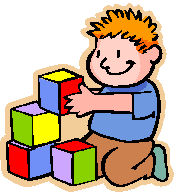 |  |
| --- | --- | --- |
| 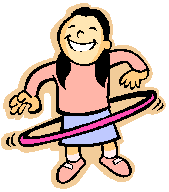 | 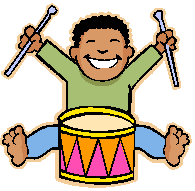 | 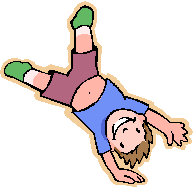 |
|  | 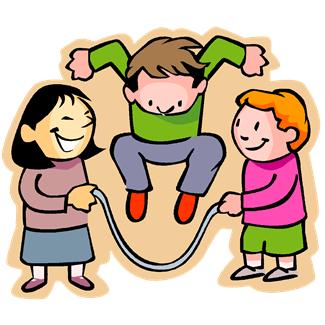 |  |

**Patch Questionnaire**

**Patch Questionnaire**

**What to do**

These questions ask you how you have felt **in the last week**. Read them all and see which one is most like you **in the last week**.

Put a tick in the box next to it, like this 🗹. Only tick one box for each question.

Last week I felt a bit angry because of my patch, so I will tick this box.

**Angry**

| I do not feel angry because of my patch ... ... ... ... ... ... ... ... ... ... ... ... ... ... ... ... ... ... ... | ...□ |
| --- | --- |
| I feel a little bit angry because of my patch ... ... ... ... ... ... ... ... ... ... ... ... ... ... ... ... ... ... | ...□ |
| I feel a bit angry because of my patch ... ... ... ... ... ... ... ... ... ... ... ... ... ... ... ... ... ... ... ... | ...🗹 |
| I feel a lot angry because of my patch ... ... ... ... ... ... ... ... ... ... ... ... ... ... ... ... ... ... ... ... | ...□ |
| I feel very angry because of my patch ... ... ... ... ... ... ... ... ... ... ... ... ... ... ... ... ... ... ... ... | ...□ |

| Now think about the questions below. |
| --- |

1. **Sad**

| My patch has not made me feel sad... ... ... ... ... ... ... ... ... ... ... ... ... ... ... ... ... ... ... ... ... | ...□ |
| --- | --- |
| My patch has made me feel a little bit sad. ... ... ... ... ... ... ... ... ... ... ... ... ... ... ... ... ... ... | ...□ |
| My patch has made me feel a bit sad. ... ... ... ... ... ... ... ... ... ... ... ... ... ... ... ... ... ... ... ... | ...□ |
| My patch has made me feel quite sad ... ... ... ... ... ... ... ... ... ... ... ... ... ... ... ... ... ... ... ... | ...□ |
| My patch has made me feel very sad.. ... ... ... ... ... ... ... ... ... ... ... ... ... ... ... ... ... ... ... ... | ...□ |

1. **Feeling of your patch on your face (like sticky, or itchy)**

| The feel of my patch has not bothered me. ... ... ... ... ... ... ... ... ... ... ... ... ... ... ... ... ... ... | ...□ |
| --- | --- |
| The feel of my patch has bothered me a little bit... ... ... ... ... ... ... ... ... ... ... ... ... ... ... ... | ...□ |
| The feel of my patch has bothered me a bit... ... ... ... ... ... ... ... ... ... ... ... ... ... ... ... ... ... | ...□ |
| The feel of my patch has bothered me quite a bit. ... ... ... ... ... ... ... ... ... ... ... ... ... ... ... | ...□ |
| The feel of my patch has bothered me a lot... ... ... ... ... ... ... ... ... ... ... ... ... ... ... ... ... ... | ...□ |
| The feel of my patch has really bothered me. ... ... ... ... ... ... ... ... ... ... ... ... ... ... ... ... ... | ...□ |

1. **Hurt**

| My patch did not hurt me.. ... ... ... ... ... ... ... ... ... ... ... ... ... ... ... ... ... ... ... ... ... ... ... ... ... | ...□ |
| --- | --- |
| My patch hurt me a little bit... ... ... ... ... ... ... ... ... ... ... ... ... ... ... ... ... ... ... ... ... ... ... ... ... | ...□ |
| My patch hurt me a bit... ... ... ... ... ... ... ... ... ... ... ... ... ... ... ... ... ... ... ... ... ... ... ... ... ... ... | ...□ |
| My patch hurt me quite a bit. ... ... ... ... ... ... ... ... ... ... ... ... ... ... ... ... ... ... ... ... ... ... ... ... | ...□ |
| My patch hurt me a lot... ... ... ... ... ... ... ... ... ... ... ... ... ... ... ... ... ... ... ... ... ... ... ... ... ... ... | ...□ |
| My patch really hurt me.. ... ... ... ... ... ... ... ... ... ... ... ... ... ... ... ... ... ... ... ... ... ... ... ... ... ... | ...□ |

1. **Doing work at school (like reading and writing)**

| My patch has not made it hard to do my work... ... ... ... ... ... ... ... ... ... ... ... ... ... ... ... ... | ...□ |
| --- | --- |
| My patch made it a little bit hard to do my work ... ... ... ... ... ... ... ... ... ... ... ... ... ... ... ... | ...□ |
| My patch made it a bit hard to do my work ... ... ... ... ... ... ... ... ... ... ... ... ... ... ... ... ... ... | ...□ |
| My patch made it quite hard to do my work.. ... ... ... ... ... ... ... ... ... ... ... ... ... ... ... ... ... | ...□ |
| My patch made it very hard to do my work ... ... ... ... ... ... ... ... ... ... ... ... ... ... ... ... ... ... | ...□ |

1. **Cross**

| My patch did not make me feel cross.. ... ... ... ... ... ... ... ... ... ... ... ... ... ... ... ... ... ... ... ... | ...□ |
| --- | --- |
| My patch made me feel a little bit cross.. ... ... ... ... ... ... ... ... ... ... ... ... ... ... ... ... ... ... ... | ...□ |
| My patch made me feel a bit cross.. ... ... ... ... ... ... ... ... ... ... ... ... ... ... ... ... ... ... ... ... ... | ...□ |
| My patch made me feel quite cross. ... ... ... ... ... ... ... ... ... ... ... ... ... ... ... ... ... ... ... ... ... | ...□ |
| My patch made me feel very cross.. ... ... ... ... ... ... ... ... ... ... ... ... ... ... ... ... ... ... ... ... ... | ...□ |

1. **How other children have treated you (like laughing at you, or calling you names) because of your patch**

| Children have not laughed at me or called me names ... ... ... ... ... ... ... ... ... ... ... ... ... | ...□ |
| --- | --- |
| Children have laughed at me or called me names a little bit.. ... ... ... ... ... ... ... ... ... ... | ...□ |
| Children have laughed at me or called me names a bit.. ... ... ... ... ... ... ... ... ... ... ... ... | ...□ |
| Children have laughed at me or called me names quite a bit ... ... ... ... ... ... ... ... ... ... | ...□ |
| Children have laughed at me or called me names a lot.. ... ... ... ... ... ... ... ... ... ... ... ... | ...□ |
| Children have really laughed at me or called me names. ... ... ... ... ... ... ... ... ... ... ... ... | ...□ |

1. **Doing things (like playing on the computer, colouring, playing games, watching TV)**

| My patch has not made it hard to do things... ... ... ... ... ... ... ... ... ... ... ... ... ... ... ... ... ... | ...□ |
| --- | --- |
| My patch has made it a little bit hard to do things. ... ... ... ... ... ... ... ... ... ... ... ... ... ... ... | ...□ |
| My patch has made it a bit hard to do things. ... ... ... ... ... ... ... ... ... ... ... ... ... ... ... ... ... | ...□ |
| My patch has made it quite hard to do things ... ... ... ... ... ... ... ... ... ... ... ... ... ... ... ... ... | ...□ |
| My patch has made it very hard to do things. ... ... ... ... ... ... ... ... ... ... ... ... ... ... ... ... ... | ...□ |

1. **Worried**

| My patch has not made me feel worried ... ... ... ... ... ... ... ... ... ... ... ... ... ... ... ... ... ... ... | ...□ |
| --- | --- |
| My patch has made me feel a little bit worried.. ... ... ... ... ... ... ... ... ... ... ... ... ... ... ... ... | ...□ |
| My patch has made me feel a bit worried.. ... ... ... ... ... ... ... ... ... ... ... ... ... ... ... ... ... ... | ...□ |
| My patch has made me feel quite worried. ... ... ... ... ... ... ... ... ... ... ... ... ... ... ... ... ... ... | ...□ |
| My patch has made me feel very worried... ... ... ... ... ... ... ... ... ... ... ... ... ... ... ... ... ... ... | ...□ |

1. **Upset**

| My patch has not made me feel upset ... ... ... ... ... ... ... ... ... ... ... ... ... ... ... ... ... ... ... ... | ...□ |
| --- | --- |
| My patch has made me feel a little bit upset.. ... ... ... ... ... ... ... ... ... ... ... ... ... ... ... ... ... | ...□ |
| My patch has made me feel a bit upset.. ... ... ... ... ... ... ... ... ... ... ... ... ... ... ... ... ... ... ... | ...□ |
| My patch has made me feel quite upset. ... ... ... ... ... ... ... ... ... ... ... ... ... ... ... ... ... ... ... | ...□ |
| My patch has made me feel very upset.. ... ... ... ... ... ... ... ... ... ... ... ... ... ... ... ... ... ... ... | ...□ |

1. **Playing with my friends**

| My patch has not stopped me playing with my friends. ... ... ... ... ... ... ... ... ... ... ... ... ... | ...□ |
| --- | --- |
| My patch has stopped me playing with my friends a little bit... ... ... ... ... ... ... ... ... ... ... | ...□ |
| My patch has stopped me playing with my friends a bit... ... ... ... ... ... ... ... ... ... ... ... ... | ...□ |
| My patch has stopped me playing with my friends quite a bit. ... ... ... ... ... ... ... ... ... ... | ...□ |
| My patch has stopped me playing with my friends a lot... ... ... ... ... ... ... ... ... ... ... ... ... | ...□ |
| My patch has really stopped me playing with my friends.. ... ... ... ... ... ... ... ... ... ... ... ... | ...□ |

1. **Happy**

| My patch has not made me feel happy.. ... ... ... ... ... ... ... ... ... ... ... ... ... ... ... ... ... ... ... | ...□ |
| --- | --- |
| My patch has made me feel a little bit happy ... ... ... ... ... ... ... ... ... ... ... ... ... ... ... ... ... | ...□ |
| My patch has made me feel a bit happy ... ... ... ... ... ... ... ... ... ... ... ... ... ... ... ... ... ... ... | ...□ |
| My patch has made feel quite happy. ... ... ... ... ... ... ... ... ... ... ... ... ... ... ... ... ... ... ... ... | ...□ |
| My patch has made me feel very happy ... ... ... ... ... ... ... ... ... ... ... ... ... ... ... ... ... ... ... | ...□ |

1. **A question for your Mummy, Daddy or whoever brought you to the hospital today**

Overall, how would you rate your child’s health over the last week?

| Excellent ... ... ... ... ... ... ... ... ... ... ... ... ... ... ... ... ... ... ... ... ... ... ... ... ... ... ... ... ... ... ... ... ... | ...□ |
| --- | --- |
| Very good. ... ... ... ... ... ... ... ... ... ... ... ... ... ... ... ... ... ... ... ... ... ... ... ... ... ... ... ... ... ... ... ... | ...□ |
| Good. ... ... ... ... ... ... ... ... ... ... ... ... ... ... ... ... ... ... ... ... ... ... ... ... ... ... ... ... ... ... ... ... ... ... | ...□ |
| Fair. ... ... ... ... ... ... ... ... ... ... ... ... ... ... ... ... ... ... ... ... ... ... ... ... ... ... ... ... ... ... ... ... ... ... ... | ...□ |
| Poor... ... ... ... ... ... ... ... ... ... ... ... ... ... ... ... ... ... ... ... ... ... ... ... ... ... ... ... ... ... ... ... ... ... ... | ...□ |

|  |  |
| --- | --- |
|  |  |
|  |  |
|  |  |
|  |  |
