# Supplementary material for: Developing the draft descriptive system for the child amblyopia treatment questionnaire (CAT-Qol): a mixed methods study
Source: Health Qual Life Outcomes. 2013 Oct 22;11:174. doi: 10.1186/1477-7525-11-174 (PMC3854484; doi:10.1186/1477-7525-11-174)
Supplement: Additional file 3 — Table S1. Modification to draft questionnaire (from beginning to end). [file 1477-7525-11-174-S3.docx]

Supplementary Material 3 Modification to draft questionnaire (from beginning to end)

| **Original** | **Modification** | **Reason** |
| --- | --- | --- |
| Subheading “Instructions” | Subheading “What to do” | Struggled to read word |
| Under “What to do”. “Read each one and all of the choices and ...” | “Read them all and....” | Less words to read, and struggled with word “choices” |
| Subheading “Example” | Removed word | Less words to read, and struggled with word |
| “Yesterday I felt a bit angry...” | “Last week I felt a bit angry...” | Struggled to read “yesterday” |
| “Feeling of drops on your face” | “Feeling of drops on your face (like stinging, or cold)” | Gave an example for each treatment to clarify question. Wording informed from original interviews with children |
| “The feel of my drops on my face have not bothered me” | “The feel of my drops have not bothered me” | Altered for each response option – less words to read |
| “The feel of my drops have not bothered me” | “The feel of my drops has not bothered me” | Altered for each response option – grammatical correction |
| “Work at school (like reading and writing)” | “Doing work at school (like reading and writing)” | More explanation for question |
| “My drops have not made it hard to do my work at school” | “My drops have not made it hard to do my work” | Less words to read |
| “How other children have treated you (like laughing at you, or calling you names)” | “How other children have treated you (like laughing at you, or calling you names) because of your drops” | More explanation for question |
| “Children have not laughed at me or called me names because of my drops” | “Children have not laughed at me or called me names” | Altered for each response option – less words to read, and able to do this without loss of clarity due to additional words in question heading |
|  |  |  |
| **Original** | **Modification** | **Reason** |
| “Doing other things (like playing on the computer, colouring, playing games, watching TV)” | “Doing things (like playing on the computer, colouring, playing games, watching TV)” | Less words to read |
| “My drops have not made it hard to do what I want to do” | “My drops have not made it hard to do things” | Less words to read |
| “How you have felt about your family (like your Mummy, Daddy, brother or sister)” | “Upset” | Result of de-briefing |
| “My drops have not made me get upset with my family” - 6-part response scale | “My drops have not made me feel upset” – 5-part response scale | Result of de-briefing, now Category B type question |
| “Playing with other children” | “Playing with my friends” | Result of de-briefing, easier to read |
| “My drops have not stopped me playing with other children” | “My drops have not stopped me playing with my friends” | Altered for each response option – less words to read |
| “My glasses, patch and drops did not hurt me” | “My glasses, patch or drops did not hurt me” | “My glasses, patch and drops did not hurt me” |
| Altered order of questions |  |  |
| Category B questions 6-part response scale | Category B questions 5-part response scale (removed “really”) | Result of ranking exercise – “very” and “really” were considered as being the same |
| Frustration question | Removed in its entirety | Children did not understand question |
|  | Added “happy” question, category B question (5-part response question) | Result of debriefing – children reported that they felt “happy” about gls |
|  | Added General Health Question to end of questionnaire | To measure parental view of child’s current general health |
